# Editorial: Digital medicine and artificial intelligence

**DOI:** 10.3389/frai.2026.1900837

**Published:** 2026-07-29

**Authors:** Mini Han Wang, Norberto Peporine Lopes, Nuno S. Osório

**Affiliations:** 1 Institute of Artificial Intelligence, Shenzhen University of Advanced Technology, Shenzhen, China; 2 Faculty of Medicine, The Chinese University of Hong Kong, Hong Kong SAR, China; 3 School of Pharmaceutical Sciences of Ribeirao Preto, University of São Paulo, Ribeirão Preto, Brazil; 4 Life and Health Sciences Research Institute (ICVS), School of Medicine, University of Minho, Braga, Portugal

**Keywords:** artificial intelligence, digital medicine, explainable artificial intelligence (XAI), mobile healthcare, precision medicine

The Research Topic “*Digital medicine and artificial intelligence*” was established to highlight emerging innovations and interdisciplinary advances at the intersection of Artificial Intelligence (AI) and healthcare. The contributions collected within this Research Topic encompass a broad range of themes, including explainable AI, AI-driven diagnostics, medical imaging analysis, multimodal data fusion, federated learning, mobile healthcare, intelligent clinical decision support systems, and precision medicine applications. Collectively, these studies demonstrate the expanding role of AI in enhancing healthcare quality, accessibility, efficiency, and personalization while also addressing important technical, ethical, and translational considerations.

As illustrated in Supplementary Table 1, the studies published in this Research Topic can be broadly organized into several major thematic research clusters, collectively reflecting the rapidly advancing and increasingly interdisciplinary nature of digital medicine and AI. The first thematic cluster focuses on AI-enabled diagnostics and medical imaging. Collectively, these studies demonstrate the growing maturity of AI-assisted diagnosis across multiple medical specialties. Rather than focusing solely on disease detection, they illustrate a transition toward quantitative image analysis (Raval et al.), explainable prediction, and clinically interpretable decision support. Representative examples include AI-assisted tuberculosis screening, ophthalmic (Wang et al., 2024a; Wang, 2025) image segmentation for meibomian gland dysfunction (Yu Z. et al.), transformer-based thyroid ultrasound segmentation (Xiang et al.), and AI applications in dermatology (Yu J. et al.). Nevertheless, external validation and prospective evaluation remain limited, highlighting important priorities for future clinical translation.

The second thematic cluster centers on digital health interventions and AI-assisted chronic disease management. The randomized controlled trial conducted by Roth et al. evaluated an app-based multimodal digital intervention for individuals with type 2 diabetes. The longitudinal study by Pujante et al. assessed one-year outcomes of automated insulin delivery systems in adults with type 1 diabetes. López-Tello et al. developed a mobile-based cognitive behavioral therapy intervention for anxiety and depression management in Mexico. These contributions collectively highlight the increasing importance of scalable, patient-centered, and real-world deployable digital healthcare technologies capable of supporting chronic disease monitoring, behavioral intervention, and personalized long-term healthcare management.

A third major research cluster involves large language models (LLMs), generative artificial intelligence, and biomedical natural language processing. The narrative review by Luo et al. examined the role of ChatGPT in rehabilitation medicine. The benchmarking study by Rossettini et al. compared the clinical guideline adherence of ChatGPT-3.5, ChatGPT-4o, Gemini, Claude, Copilot, and Perplexity for lumbosacral radicular pain recommendations. Souto et al. conducted a longitudinal evaluation assessing ChatGPT performance on medical residency examinations. The study by Veintimilla et al. applied AI language models to extract ancestry information from curated biomedical literature. Collectively, these studies underscore both the transformative potential and current limitations of generative AI in healthcare, particularly regarding reliability, interpretability, hallucination risks, benchmark evaluation, and clinical safety.

Another important thematic cluster addresses healthcare systems, implementation science, and human-centered AI. The review by Vernic et al. discussed the digital transformation of intensive care units. Mohajer-Bastami et al. conducted a multidisciplinary review discussing the applications, challenges, and future directions of AI in healthcare. The survey-based implementation study by Schulz et al. investigated clinician trust of and risk perception toward medical AI adoption. An expertise graph-based healthcare workforce matching framework was proposed by Xiao et al. Krumsvik and Slettvoll conducted a rural healthcare accessibility study to explore AI-supported health empowerment in geographically isolated communities. Together, these studies emphasize that the successful implementation of medical AI depends not only on technological innovation but also on clinician trust, ethical governance, healthcare accessibility, infrastructure readiness, and effective integration into real-world healthcare ecosystems.

Finally, several studies contributed to the thematic cluster of methodological and infrastructural innovations for medical AI. An adaptive noise-augmented attention framework for transformer fine-tuning on longitudinal medical data was proposed by Amirahmadi et al. and a search-optimized quantization framework for biomedical ontology alignment was developed by Bouaggad and Grabar. These methodological advances aim to improve the robustness, interoperability, scalability, and computational efficiency of healthcare AI systems, thereby facilitating the development of more reliable, efficient, and clinically deployable intelligent healthcare infrastructures. Overall, the thematic clusters identified within this Research Topic collectively demonstrate how digital medicine and AI are evolving toward increasingly interdisciplinary, multimodal, explainable, and clinically translatable healthcare ecosystems capable of advancing disease diagnosis, treatment optimization, precision medicine, and public health management.

In conclusion, this Research Topic provides a broad and interdisciplinary overview of current advances in AI-driven healthcare research and highlights the growing integration of digital technologies into modern medical practice. The published contributions collectively demonstrate the potential of AI to support disease diagnosis, medical imaging, chronic disease management, rehabilitation, biomedical informatics, digital therapeutics, and public health applications. In particular, the studies included in this Research Topic present a diverse range of emerging approaches, including deep learning, transformer architectures, large language models, generative AI, mobile health technologies, and intelligent healthcare systems, reflecting the increasingly multidisciplinary nature of digital medicine. At the same time, several contributions emphasize important translational and practical considerations, such as explainability, clinical validation, ethical governance, healthcare accessibility, clinician trust, and real-world implementation, which remain essential for the responsible deployment of AI in healthcare settings.

Although significant progress has been made, the studies presented in this Research Topic also highlight that many challenges remain, including issues related to reliability, interpretability, data privacy, fairness, benchmark standardization, and regulatory oversight. Nevertheless, the collective findings suggest that digital medicine and artificial intelligence continue to hold substantial promise for improving healthcare efficiency, personalization, and accessibility. We hope that the articles included will contribute to ongoing interdisciplinary dialogue and inspire further research toward the development of trustworthy, clinically meaningful, and patient-centered AI-enabled healthcare systems. Ultimately, continued collaboration among clinicians, researchers, engineers, ethicists, and policymakers will be essential to ensuring the responsible and sustainable advancement of AI in medicine and public health.

**Figure 1:**
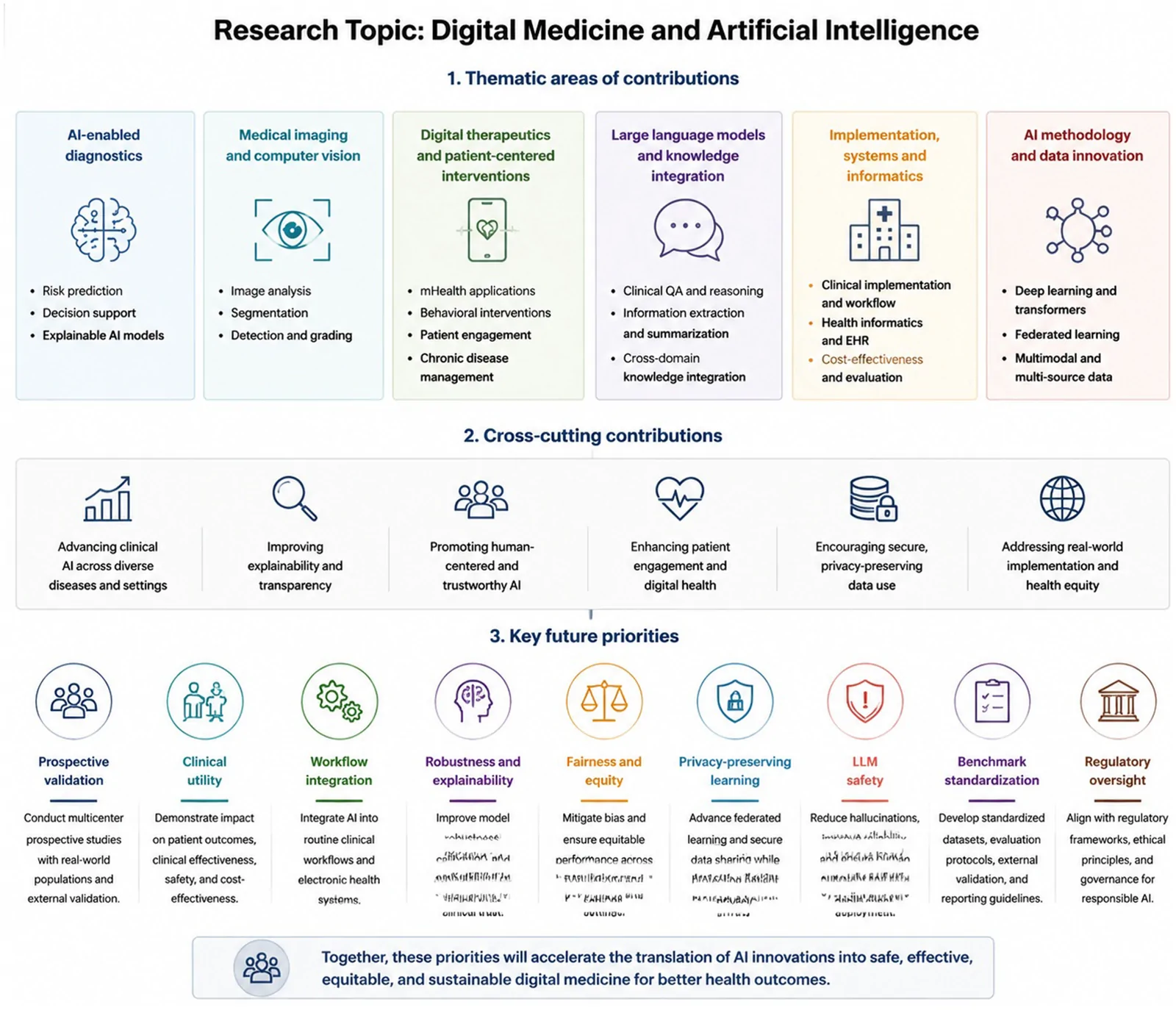
Collective contributions to and future priorities for the Research Topic “*Digital medicine and artificial intelligence*.”

The contributions collected in this Research Topic collectively indicate that the next phase of digital medicine should focus less on algorithmic novelty and more on generating clinically actionable evidence that supports routine healthcare implementation (Figure 1). Although the included studies demonstrate substantial advances across AI-enabled diagnostics, digital therapeutics, medical imaging, large language models, implementation science, and healthcare informatics, they also reveal several common priorities that should guide future research. Building upon these advances, future innovation is expected to further benefit from emerging technologies that complement the themes represented in this Research Topic, including mobile health (mHealth) (Wang et al., 2025), LLMs, generative artificial intelligence (Generative AI), wearable sensing systems (Song et al., 2026), federated learning (Fu et al., 2026), and multimodal foundation models (Wang et al., 2024b). However, their successful translation into clinical practice will depend not only on technological innovation but also on rigorous prospective validation, demonstrated clinical utility, seamless workflow integration, fairness, privacy-preserving learning, LLM safety, benchmark standardization, and appropriate regulatory oversight.

First, prospective, multicenter, and real-world validation should become the standard for evaluating medical AI systems. Many current studies demonstrate encouraging technical performance, but evidence regarding clinical effectiveness, generalizability across healthcare settings, and long-term patient outcomes remains limited. Future work should therefore emphasize prospective evaluation, external validation, and assessment of clinical utility rather than relying primarily on retrospective benchmark datasets.

Second, successful translation will require seamless integration of AI into existing clinical workflows. Several studies within this Research Topic highlight that clinician acceptance, usability, implementation strategy, and healthcare infrastructure are equally important determinants of adoption. Future research should therefore prioritize human-centered system design, workflow integration, and outcome measures that reflect improvements in healthcare delivery, efficiency, and patient care.

Third, trustworthy AI should remain a central objective for digital medicine. The growing application of LLMs and foundation models offers considerable opportunities for clinical decision support, medical education, and biomedical knowledge synthesis, while simultaneously raising important concerns regarding hallucinations, bias, explainability, transparency, and accountability. Future studies should place greater emphasis on safety evaluation, human oversight, and clinically meaningful benchmark development for LLM-based healthcare applications.

In parallel, privacy-preserving and collaborative learning frameworks will become increasingly important as healthcare data continue to be distributed across institutions and jurisdictions. Federated learning and related privacy-enhancing technologies offer promising approaches for developing robust and generalizable AI systems while respecting patient confidentiality and regulatory requirements.

Finally, the collective findings of this Research Topic underscore the need for standardized evaluation frameworks that extend beyond conventional performance metrics. Future research should incorporate benchmark standardization, fairness assessment, calibration, robustness, reproducibility, and clinically relevant outcome measures alongside closer alignment with emerging regulatory and ethical frameworks. Addressing these priorities will help facilitate the responsible translation of AI from proof-of-concept research to safe, effective, and sustainable clinical practice.
