# Correction: Editorial: Digital medicine and artificial intelligence

**DOI:** 10.3389/frai.2026.1965611

**Published:** 2026-09-03

**Authors:** Mini Han Wang, Norberto Peporine Lopes, Nuno S. Osório

**Affiliations:** 1 Institute of Artificial Intelligence, Shenzhen University of Advanced Technology, Shenzhen, China; 2 Faculty of Medicine, The Chinese University of Hong Kong, Hong Kong SAR, China; 3 School of Pharmaceutical Sciences of Ribeirao Preto, University of São Paulo, Ribeirão Preto, Brazil; 4 Life and Health Sciences Research Institute (ICVS), School of Medicine, University of Minho, Braga, Portugal

**Keywords:** artificial intelligence, digital medicine, explainable artificial intelligence (XAI), mobile healthcare, precision medicine

The reference for Chen et al. (2026) was erroneously cited in paragraph 9. The citation along with the reference “Chen, K.-Y., Chan, H.-C., Hwang, Y.-S., Lin, W.-W., and Chan, C.-M. (2026). Are Müller glial cells gatekeepers of neuroprotection and regeneration in age-related macular degeneration? Unraveling their roles in pathophysiology and therapeutic innovation. *Prog. Retin. Eye Res*. 112:101471. doi: 10.1016/j.preteyeres.2026.101471” will be removed.

A correction has been made to Paragraph 9. The paragraph will now read:

“The contributions collected in this Research Topic collectively indicate that the next phase of digital medicine should focus less on algorithmic novelty and more on generating clinically actionable evidence that supports routine healthcare implementation (Figure 1). Although the included studies demonstrate substantial advances across AI-enabled diagnostics, digital therapeutics, medical imaging, large language models, implementation science, and healthcare informatics, they also reveal several common priorities that should guide future research. Building upon these advances, future innovation is expected to further benefit from emerging technologies that complement the themes represented in this Research Topic, including mobile health (mHealth) (Wang et al., 2025), LLMs, generative artificial intelligence (Generative AI), wearable sensing systems (Song et al., 2026), federated learning (Fu et al., 2026), and multimodal foundation models (Wang et al., 2024b). However, their successful translation into clinical practice will depend not only on technological innovation but also on rigorous prospective validation, demonstrated clinical utility, seamless workflow integration, fairness, privacy-preserving learning, LLM safety, benchmark standardization, and appropriate regulatory oversight.”

The original version of this article has been updated.

