# Supplementary material for: Editorial: Digital medicine and artificial intelligence
Source: Front Artif Intell. 2026 Jul 29;9:1900837. doi: 10.3389/frai.2026.1900837 (PMC13461700; doi:10.3389/frai.2026.1900837)
Supplement: Supplementary file 1 [file Table_1.docx]

| **Study (Author, Year)** | **Study Type** | **Methods** | **Novelty / Contribution** | **Main Findings** | **Strengths** |
| --- | --- | --- | --- | --- | --- |
| Roth et al., 2025[7] | Clinical Trial | Randomized controlled trial of an app-based multimodal intervention for type 2 diabetes | Compared a therapeutic digital intervention with a placebo application | Digital interventions improved diabetes self-management and demonstrated feasibility in chronic disease care | Strong clinical trial design and real-world translational value |
| Raval et al., 2025[1] | Original Research | Rapid health technology assessment and cost-effectiveness analysis of AI-assisted chest X-ray interpretation | Integrated economic evaluation with AI-based tuberculosis screening | AI-assisted chest X-ray interpretation demonstrated potential cost-effectiveness and screening utility | Strong public health and healthcare policy relevance |
| Vernic et al., 2025[14] | Review | Narrative review of ICU digital transformation | Synthesized the impact of digital technologies and AI in intensive care units | Digital systems may improve ICU workflow efficiency, monitoring, and patient outcomes | Broad systems-level critical care perspective |
| Mohajer-Bastami et al., 2025[15] | Review | International multidisciplinary narrative review | Comprehensive overview of AI applications, challenges, and future healthcare directions | AI has transformative potential but faces interpretability, ethical, and regulatory barriers | Highly interdisciplinary and forward-looking review |
| Yu et al., 2025[4] | Original Research | Deep learning-based infrared image segmentation | Quantitative evaluation of meibomian gland dysfunction using ophthalmic imaging AI | Automated segmentation enabled objective assessment of gland morphology and dysfunction | Clinically relevant ophthalmic AI application |
| Luo et al., 2025[10] | Review | Narrative review of ChatGPT in rehabilitation medicine | Evaluated the role of large language models in rehabilitation workflows | ChatGPT showed potential in education, communication, and clinical support, but validation is still needed | Timely exploration of generative AI in rehabilitation medicine |
| Bouaggad and Grabar, 2025[20] | Original Research | Search-optimized quantization for biomedical ontology alignment | Improved semantic matching efficiency in biomedical ontologies | Enhanced ontology alignment performance with computational optimization | Strong methodological contribution to biomedical informatics |
| López-Tello et al., 2025[9] | Original Research | Mobile-based cognitive behavioral therapy intervention | Digital CBT platform targeting anxiety and depression in Mexico | Mobile intervention demonstrated feasibility for mental health management | Important contribution to digital mental health accessibility |
| Veintimilla et al., 2025[13] | Original Research | AI language models for biomedical literature mining | Automated ancestry information extraction from curated biomedical literature | AI models improved structured extraction of ancestry-related biomedical information | Valuable application of biomedical NLP and AI text mining |
| Amirahmadi et al., 2025[19] | Methods | Adaptive noise-augmented attention for Transformer fine-tuning | Enhanced Transformer learning for longitudinal medical data | Improved representation learning and robustness in clinical temporal data | Advanced methodological innovation for healthcare AI |
| Xiao et al., 2025[17] | Original Research | Industrial engineering expertise graph | Intelligent matching between learning ability and healthcare workforce demand | Graph-based AI improved educational and healthcare workforce alignment | Novel application of AI in healthcare education and workforce systems |
| Schulz et al., 2025[16] | Original Research | Survey-based implementation research | Investigated trust and risk perception in medical AI adoption | Clinical experience and perceived risk strongly influenced AI acceptance | Important human-centered and implementation science perspective |
| Krumsvik and Slettvoll, 2025[18] | Brief Research Report | Case-based digital health analysis | AI-supported health empowerment in geographically isolated communities | AI technologies may improve healthcare accessibility in rural or disaster-isolated settings | Strong public health and accessibility implications |
| Souto et al., 2025[12] | Brief Research Report | Longitudinal benchmarking of ChatGPT on residency examinations | Multi-model evaluation of LLM performance over time | LLM performance improved but remained variable across medical tasks | Valuable benchmark-style assessment of generative AI in medicine |
| Yu et al., 2025[6] | Systematic Review | Systematic review of AI in dermatology in China | Summarized AI applications, datasets, and challenges in dermatology | AI demonstrated promise in dermatological diagnosis and image analysis | Comprehensive disease-specific synthesis |
| Xiang et al., 2025[5] | Original Research | Transformer models with masked autoencoder pre-training | Self-supervised pre-training for thyroid ultrasound segmentation | Improved thyroid nodule segmentation accuracy and robustness | Advanced transformer-based medical imaging methodology |
| Pujante et al., 2025[8] | Original Research | One-year clinical evaluation of automated insulin delivery systems | Longitudinal assessment of AI-supported diabetes management systems | Automated insulin delivery improved glycemic management in type 1 diabetes | Strong real-world clinical applicability |
| Rossettini et al., 2025[11] | Original Research | Cross-sectional benchmarking study of ChatGPT, Gemini, Claude, Copilot, and Perplexity | Compared multiple LLMs against clinical practice guidelines | Significant variability existed in guideline adherence and medical reasoning accuracy | Highly relevant evaluation of trustworthy clinical LLM deployment |
